# SPorDyn: A Python code for modeling the evolution of soil pore size distribution after tillage

**DOI:** 10.1016/j.mex.2019.09.014

**Published:** 2019-09-13

**Authors:** Parvathy Chandrasekhar, Janis Kreiselmeier, Andreas Schwen, Thomas Weninger, Stefan Julich, Karl-Heinz Feger, Kai Schwärzel

**Affiliations:** a United Nations University Institute for Integrated Management of Material Fluxes and of Resources (UNU-FLORES), Ammonstraße 74, 01067 Dresden, Germany; b Institute of Soil Science and Site Ecology, Technische Universität Dresden, Pienner Straße 19, 01737 Tharandt, Germany; c Institute for Soil Physics and Rural Water Management, University of Natural Resources and Life Sciences, Muthgasse 18, 1190 Vienna, Austria; d Institute of Forest Ecosystems, Thünen Institute, Alfred-Möller Straße 1, 16225 Eberswalde Germany

**Keywords:** SPorDyn: Soil pore dynamics using Python, Pore evolution model, Soil structure, Temporal dynamics, Tillage

## Abstract

Surface soil structure is very responsive to natural and anthropogenic impacts and these changes alter soil hydraulic properties and the soil water budget. In the midst of a dearth of efforts to capture soil structural dynamics, an analytical solution to the Fokker-Planck Equation with physically-based coefficients has shown promising results in predicting the evolution of soil pore space in agricultural soils. In this study, the Python code for the analytical solution is shown along with steps to estimate coefficients leading towards obtaining the analytical solution.

• Python code for modeling the evolution of soil pore space based on an existing model is shared.
• The code for the estimation of physically-based coefficients of the model and parameter optimization are also shown.
• The final output of the model may be used in estimation of soil water retention and hydraulic conductivity functions.

## Graphical abstract

**Figure:**
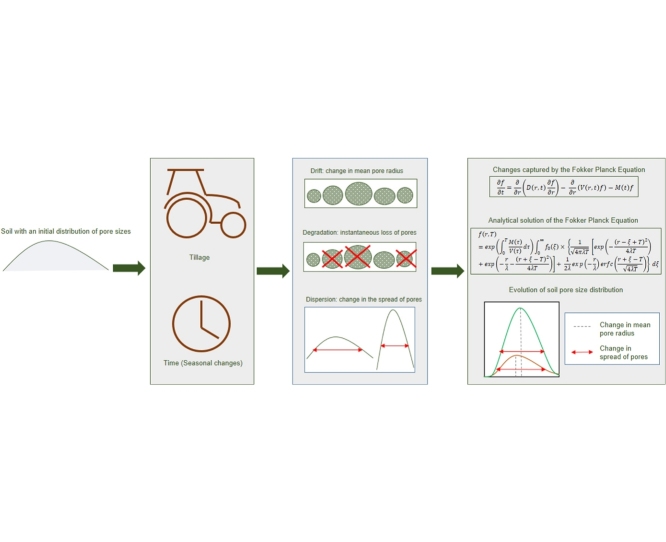

**Specification Table**

**Table:** 

Subject Area:	• *Agricultural and Biological Sciences* • *Environmental Science*
More specific subject area:	*Modeling approach to capture the temporal dynamics of soil pore space*
Method name:	*SPorDyn: Soil pore dynamics using Python*
Name and reference of original method:	*Or, D., Leij, F.J., Snyder, V., Ghezzehei, T.A., 2000. Stochastic model for posttillage soil pore space evolution. Water Resour. Res. 36, 1641–1652.* *Leij, F.J., Ghezzehei, T.A., Or, D., 2002a. Modeling the dynamics of the soil pore-size distribution. Soil Tillage Res. 64, 61–78.* *Leij, F.J., Ghezzehei, T.A., Or, D., 2002b. Analytical models for soil pore-size distribution after tillage. Soil Sci. Soc. Am. J. 66, 1104–1114.* *Chandrasekhar,P., Kreiselmeier, J., Schwen,A., Weninger,T., Julich,S., Feger, K.-H., Schwärzel, K., 2019. K. Modeling the evolution of soil structural pore space in agricultural soils following tillage. Geoderma. 353. 401 - 414*
Resource availability:	*Code provided as supplementary material and as images in the text*

## Method details

Surface soil structure is responsive to both natural and anthropogenic changes and consequently impacts soil hydraulic properties (SHP). Generally, the structural pore space with pore radius (*r*) ≥ 5 μm is expected to be the most affected by management practices such as tillage and natural stresses (e.g. rainfall). The need for inclusion of soil pore dynamics in hydrological models to improve their reliability and accuracy has been stressed in recent times [[1], [2], [3]]. In this context, Or et al. [4] proposed to use a partial differential equation (PDE) known as the Fokker-Planck Equation to capture the dynamics of soil pore size distribution (PSD) following tillage with respect to time and pore radius. Knowledge of the PSD will pave way to predict unsaturated SHP which can then be incorporated in hydrological models. The PDE comprises physically-based coefficients (drift (*V*), degradation (*M*) and dispersion (*D*) coefficients) and they encompass our perception of the mathematical behavior of soil PSD in response to tillage practices. The coefficients are subject to an initial condition as well as upper and lower boundary conditions. Based on these conditions, an analytical solution to the proposed PDE was provided by Leij et al. [5,6]. In Chandrasekhar et al. [1], the model was applied to different water retention parameter data sets around the world to evaluate its capability in predicting the temporal dynamics of soil pore space for two cases (1) when there is a change in the tillage regime/land-use and (2) in the months following tillage. In the present contribution, we share the Python code used in Chandrasekhar et al. [1] to capture the evolution of soil pore space following tillage.

This paper is organized as follows: As a first step, a skeletal framework of the mathematical model is briefly described. For a detailed overview of the model and its application, the reader is directed to Chandrasekhar et al. [1]. The required input data is listed. Brief descriptions of the remaining aspects of the model such as the coefficients as well as the accompanying code are then provided. Finally, the optimization process and steps to obtain the analytical solution are given.

## Mathematical model

In this section, a skeletal overview of the mathematical model and its coefficients is provided. The following partial differential equation (PDE), also known as the Fokker-Planck equation, is used to describe the evolution of soil PSD following tillage [4]:

(1) $$\frac{\partial f}{\partial t}=\frac{\partial }{\partial r}\left(D\left(r,t\right)\frac{\partial f}{\partial r}\right)-\;\frac{\partial }{\partial r}\left(V\left(r,t\right)f\right)-M\left(t\right)f$$

where *f* is the PSD or frequency [L^−1^] of pores as a function of time *t* [T] and pore radius *r* [L], *D* the dispersion coefficient [L^2^ T^−1^], *V* the drift coefficient [LT^−1^] and *M* the degradation coefficient [T^−1^]. *D* and *V* quantify the changes with time of the variance of the PSD and mean pore radius, respectively, while *M* is a first-order degradation factor representing instantaneous pore loss, i.e., the fraction of pores that are lost due to instantaneous collapse. The initial and boundary conditions for solving Eq. (1) are:

(2) $$f\left(r,0\right)=f_{0}\left(r\right),\;0<r<\text{∞}\;$$

(3) $$Vf-D\frac{\partial f}{\partial r}=0,\;r=0,\;t>0$$

(4) $$\frac{\partial f}{\partial r}=0,\;r\to \text{∞},\;t>0$$

*f*_0_ in Eq. (2) is the initial PSD for the parameterization of which the lognormal distribution function of [7] is used. The lower boundary condition (Eq. 3) stipulates a zero-probability flux meaning that only positive pore sizes are allowed while the upper boundary condition (Eq. 4) necessitates a zero gradient for infinitely large pore radii. The PDE subject to the initial and boundary conditions yields the following analytical solution [5,6]:

(5) $$f\left(r,T\right)=exp\left(\int_{0}^{T}\frac{M\left(\tau \right)}{V\left(\tau \right)}d\tau \right)\int_{0}^{\text{∞}}f_{0}\left(\xi \right)\times \;\left\{\frac{1}{\sqrt{4\pi \lambda T}}\;\left[exp\left(-\frac{{\left(r-\xi +T\right)}^{2}}{4\lambda T}\right)+exp\left(-\frac{r}{\lambda }-\frac{{\left(r+\xi -T\right)}^{2}}{4\lambda T}\right)\right]+\;\frac{1}{2\lambda }exp\left(-\frac{r}{\lambda }\right)erfc\left(\frac{r+\xi -T}{\sqrt{4\lambda T}}\right)\right\}\;d\xi \;$$

where *τ* and *ξ* are dummy integration variables. Due to the fact that tillage treatment cannot be readily converted to time as an independent variable, the cumulative drift term *T* is used as an independent variable instead of time, meaning that the evolution of PSD is predicted based on the gradual changes in the pore radii [5].

## Input data

The input data for the modeling approach are water retention parameters obtained at different temporal stages. These parameters may be parameterized according to the Kosugi [7] water retention model. If Van Genuchten parameters [8] are available, they can be converted to the Kosugi parameters which is explained in Chandrasekhar et al. [1]. The required input water retention parameters are listed in Table 1.

**Table 1:** Input data: water retention parameters.

Parameter	Unit	Description
*θ_s_*	[L^3^ L^−3^]	Saturated water content
*θ_r_*	[L^3^ L^−3^]	Residual water content
*σ*	[-]	Standard deviation of the log-transformed pore radius
*r_m_*	[L]	Median pore radius

## Initial pore size distribution

**Fig. 1:**
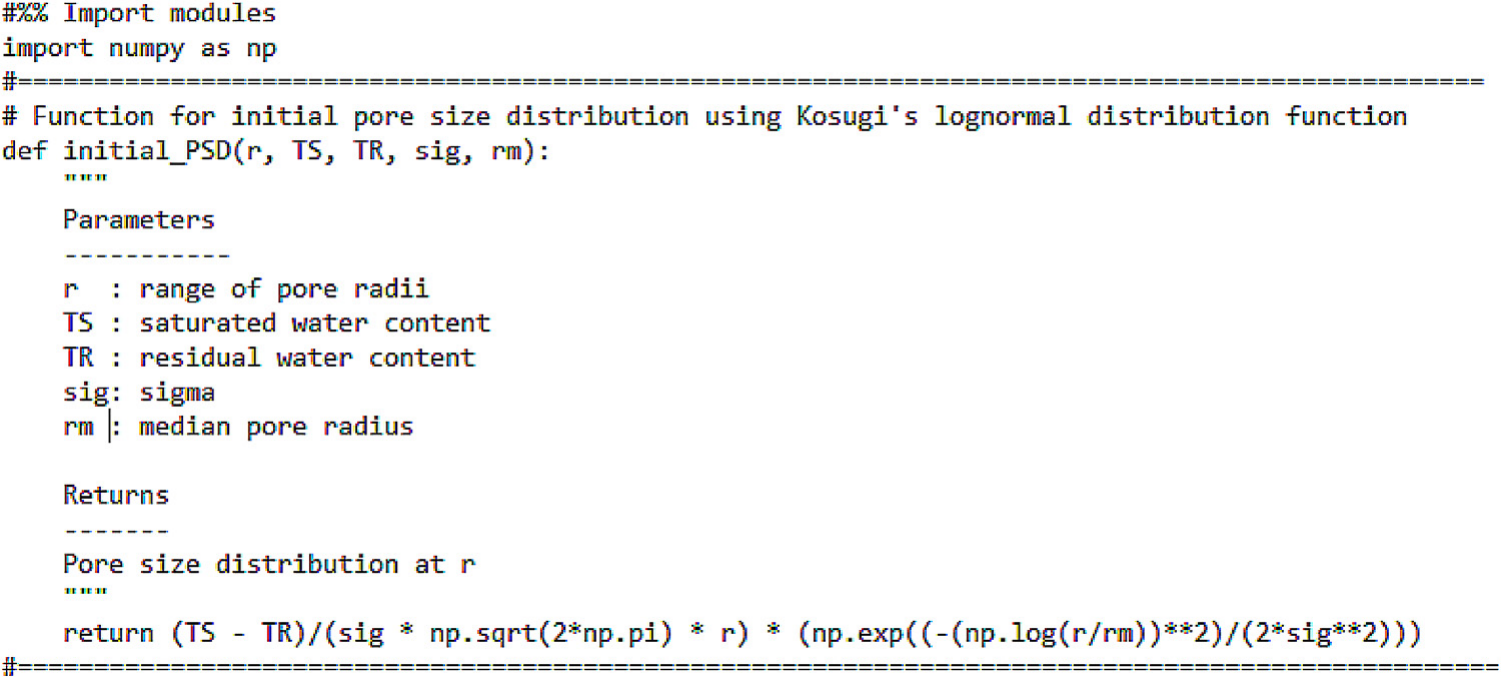
Code for initial pore size distribution.

Eq. (6) shows the lognormal distribution function for the pore radii [7]:

(6) $$f_{0}\left(r\right)=\;\frac{\phi_{0}}{r\sigma \sqrt{2\pi }}\exp\left(-\frac{{\left[\ln\left(\frac{r}{r_{m}}\right)\right]}^{2}}{2\sigma^{2}}\right)$$

$$where\int_{0}^{\text{∞}}f_{0}\left(r\right)dr=\;\phi_{0},\;0<r<\;\text{∞}\;$$

where *ϕ*_0_ [-] is the total initial porosity, *r*_m_ [L] is the initial median pore radius or geometric mean and *σ* [-] is the standard deviation of the log-transformed pore radius. Leij et al. [5] assumed *ϕ*_0_ to be the difference between saturated and residual water contents: *ϕ*_0_ = *θ*_s_ – *θ*_r_. Finally, r_m_ is calculated from the Young-Laplace equation where r = A/h, where A is a proportionality constant obtained from the variables of the equation, A = −0.149 cm^2^ and h [L] is the pressure head. The code for the initial PSD is shown in Fig. 1.

## Coefficients of the PDE

The coefficients of the PDE are calculated following the approach of [4] who used moment analysis of the PSD to yield the definitions for mean and variance. Moments are defined by integrating the PSD with respect to the pore size:

(7) $$m_{n}\left(T\right)=\int_{0}^{\text{∞}}r^{n}\;f\left(r,T\right)dr,\;n=0,1,2$$

**Fig. 2:**
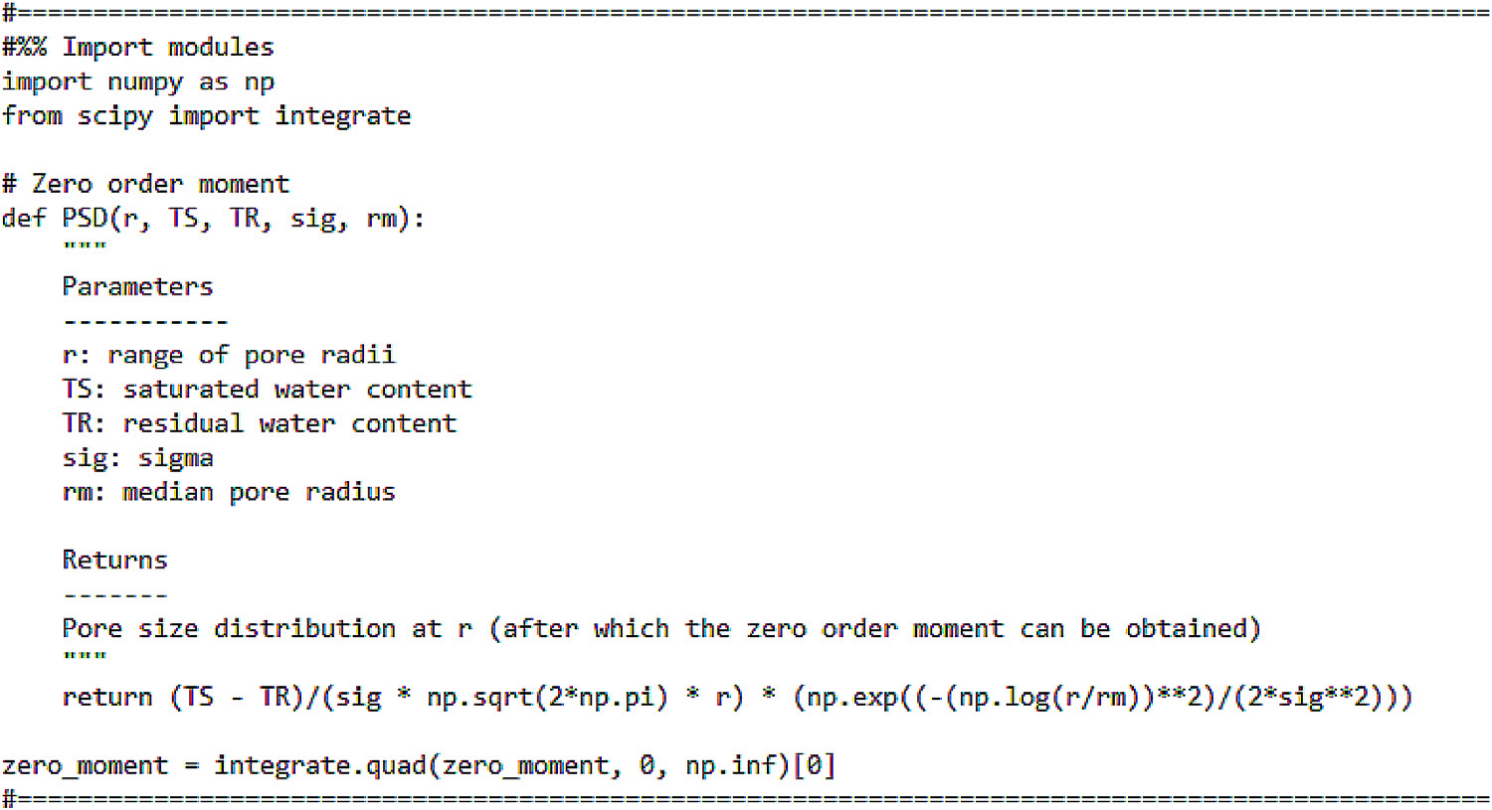
Code for zero-order moment.

Normalized moments (M_n_) are calculated through division by the zero-order moment (m_0_). The code to obtain the zero order moment is shown in Fig. 2.

## Degradation term

The degradation term is expressed by means of an exponentially decaying function [9]:

(8) $$M\left(t\right)=d\times \text{exp}\left(ct\right),\;c<0$$

**Fig. 3:**
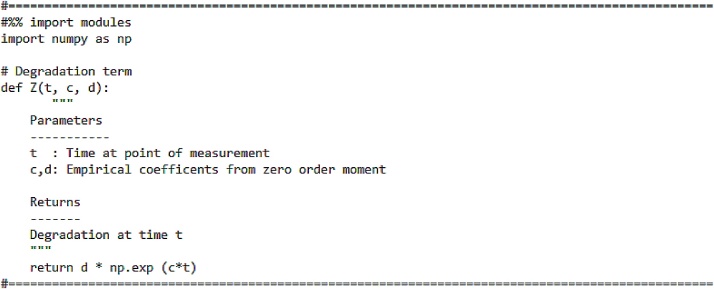
Code for degradation term.

Here, c and d are empirical coefficients which are obtained from the zero-order moment. In our study using moment analysis, we evaluated *m_0_* values to check if the probability was preserved. If the probability was not preserved, we included the degradation term in the analytical solution. The code for the degradation term is shown in Fig. 3.

## Drift term

The first-order normalized moment M_1_ characterizes the mean pore size 〈r〉 [L]:

(9) $$M_{1}=\;<r>\;=\;r_{m}\;\text{e}\text{x}\text{p}\left(\frac{\sigma^{2}}{2}\right)\;$$

The first-order moment can also be obtained from other independent models [1]. For instance, the rather popular expression from Thornley [10] is used for the drift term.

(10) $$V\left(t\right)=\;\frac{d}{dt}\langle r\rangle =a\left(1-\;\frac{\langle r\rangle }{b}\right)\langle r\rangle \;where\;\langle r\rangle =\;\frac{b\langle r_{0}\rangle }{\langle r_{0}\rangle +\left(b-\;\langle r_{0}\rangle \right)\text{exp}\left(-at\right)}\;$$

**Fig. 4:**
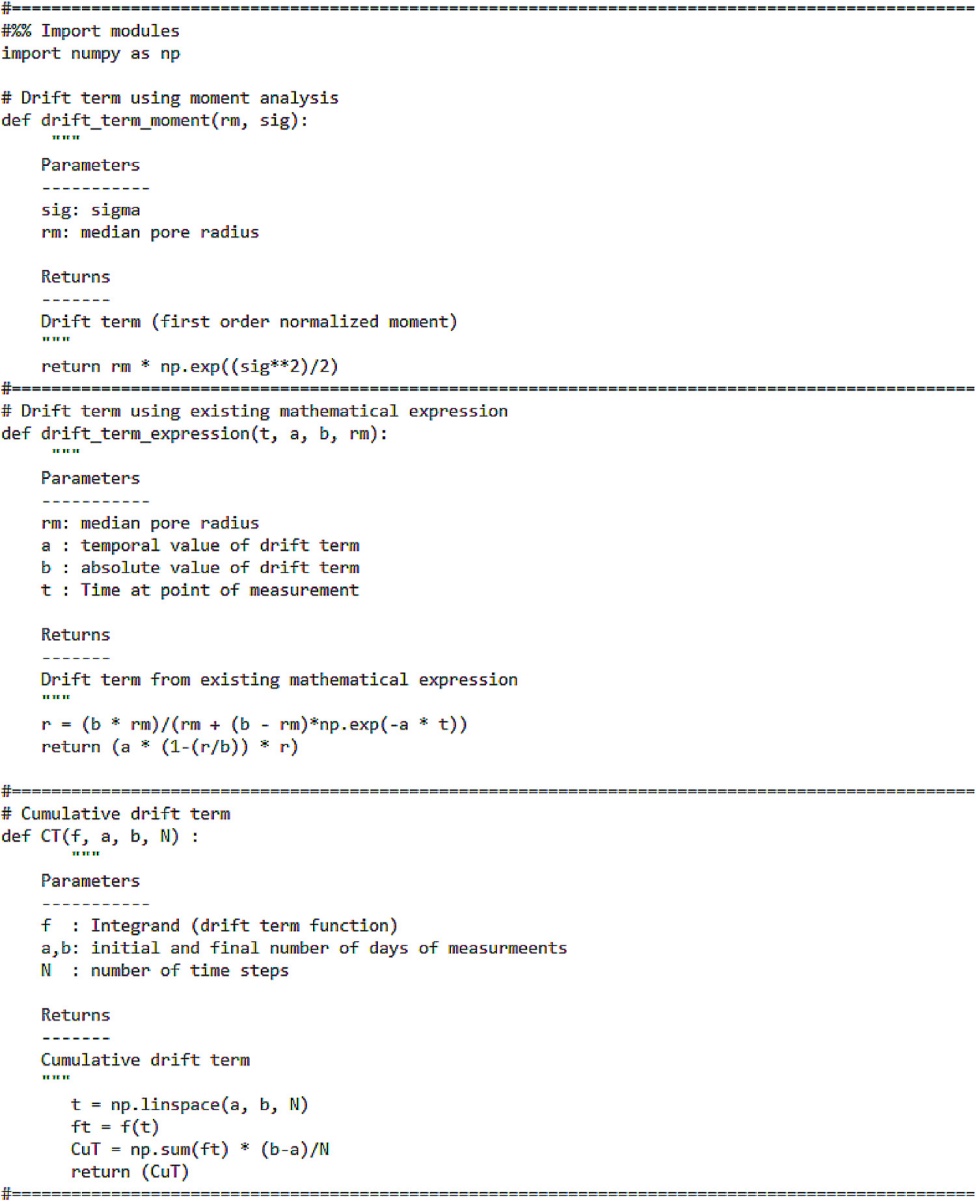
Code for drift term.

The code for both these scenarios are shown in Fig. 4.

## Dispersion term

Finally, the second-order centralized moment μ_2_ characterizes the variance [11]:

(11) $$\mu_{2}=r_{m}^{2}\exp\left(\sigma^{2}\right)\left[\exp\left(\sigma^{2}\right)-1\right]$$

However, our lack of knowledge on how the dispersion behaves with respect to the pore size paved the way to use the dispersivity *λ.*

(12) $$\lambda =\frac{D(t)}{\left|V(t\right|}\;\;$$

*λ* is obtained by fitting Eq. (5) to the observed values by means of the Levenberg-Marquardt method

## Analytical solution

**Fig. 5:**
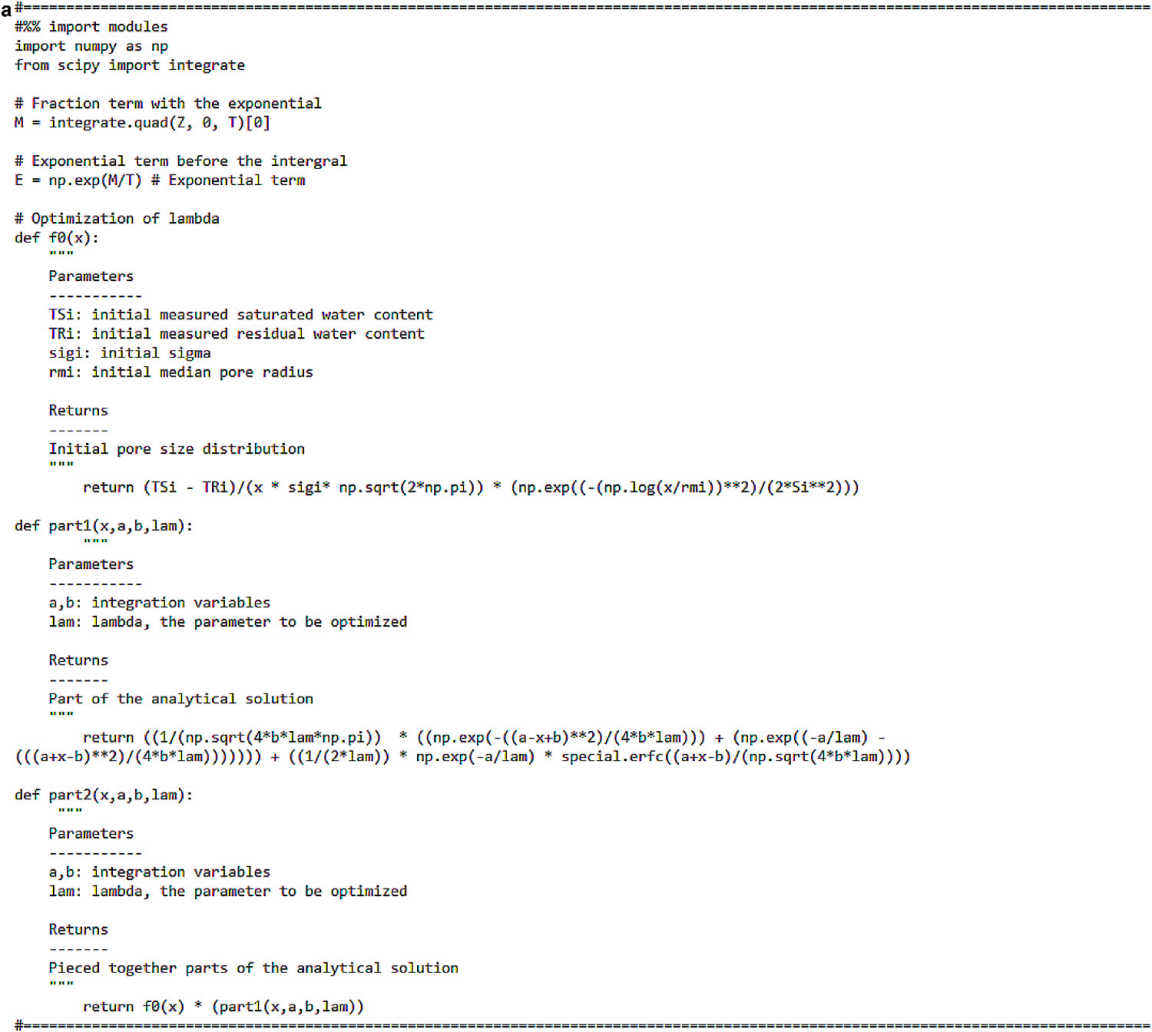

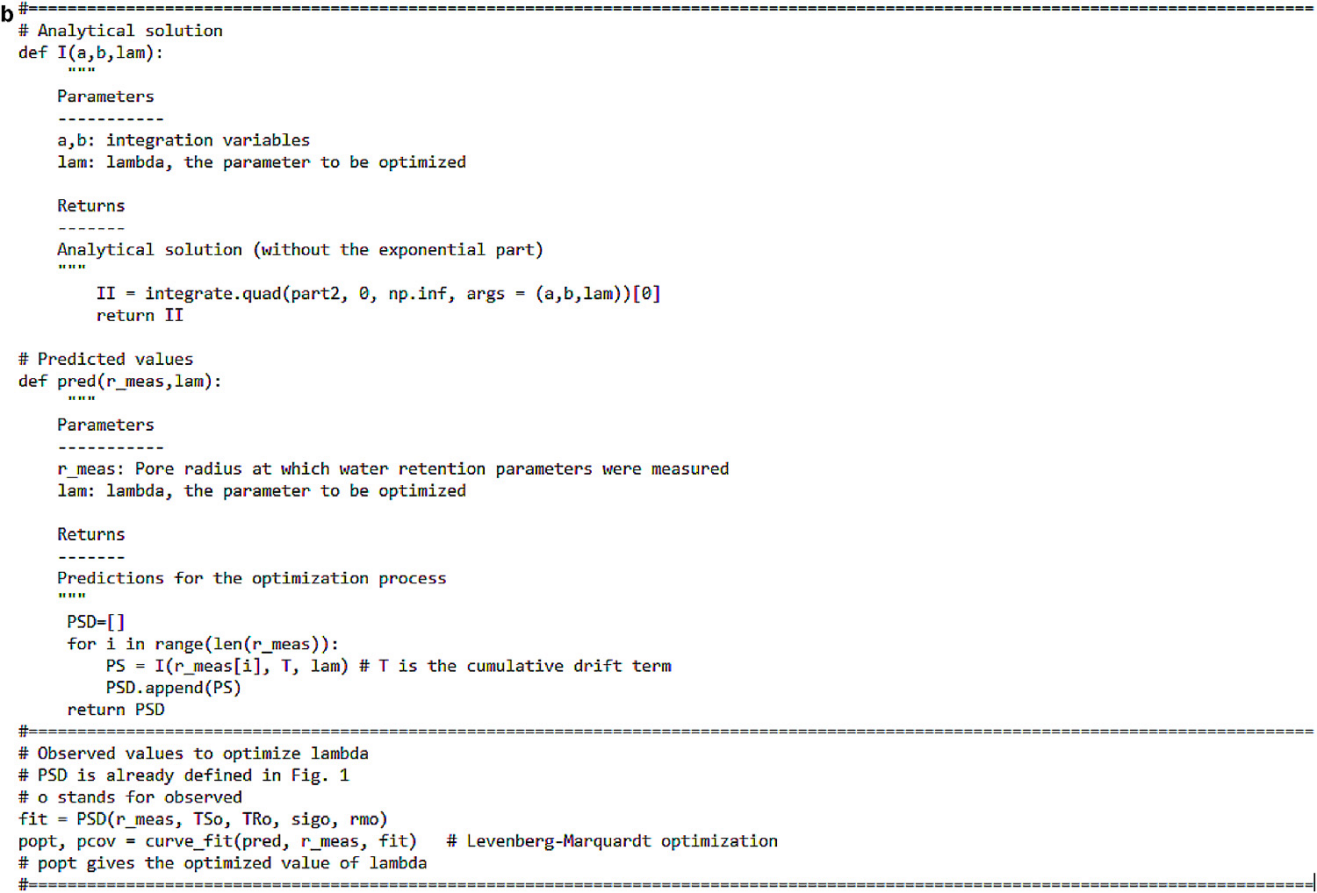
a) Part 1 of the code for optimizing lambda (and obtaining the analytical solution). b) Part 2 of the code for optimizing lambda (and obtaining the analytical solution).

As a final step, the pore size distribution (analytical solution in Eq. 5) is obtained using the code in Fig. 5a and b.

The optimized lambda value should now be used in the code (Fig. 5a and b) instead of lam to get the predicted pore size distribution curves. The reader is directed to Chandrasekhar et al. [1] for the output curves.

## Outlook

The possibility of using other independent models for the coefficients exists and is being looked into. Further, the optimization process for lambda necessitates the final water retention parameter values making the model redundant. However, lack of sufficient data sets that capture the temporal dynamics of soil structure hampers our quest to establish a range of values for the coefficients as well as for the validation process. In conclusion, the model is able to capture very well the evolution of soil pore size distribution in its current state with scope for improvement in how we estimate the coefficients.
