# Supplementary material for: SPorDyn: A Python code for modeling the evolution of soil pore size distribution after tillage
Source: MethodsX. 2019 Sep 13;6:2118–26. doi: 10.1016/j.mex.2019.09.014 (PMC6812319; doi:10.1016/j.mex.2019.09.014)
Supplement: Supplementary file 1 [file mmc1.docx]

**Supplementary material**

**Appendix A: Python code for initial pore size distribution**

# Import modules

import numpy as np

# Function for initial pore size distribution using Kosugi's lognormal distribution function

def initial_PSD(r, TS, TR, sig, rm):

""

Parameter

-----------------------------------------------------------------------------------------------------------------------------------------------------------

r: range of pore radii

TS: saturated water content

TR: residual water content

sig: sigma

rm: median pore radius

Returns

----------------------------------------------------------------------------------------------------------------------------------------------------------- Pore size distribution at r

"""

return (TS - TR)/(sig * np.sqrt(2*np.pi) * r) * (np.exp((-(np.log(r/rm))**2)/(2*sig**2)))

#========================================================================================

**Appendix B: Python code for zero order moment**

# Import modules

import numpy as np

from scipy import integrate

# 1. Code to obtain zero order moment

def PSD(r, TS, TR, sig, rm):

"""

Parameters

-----------------------------------------------------------------------------------------------------------------------------------------------------------

r: range of pore radii

TS: saturated water content

TR: residual water content

sig: sigma

rm: median pore radius

Returns

-----------------------------------------------------------------------------------------------------------------------------------------------------------

Pore size distribution at r (after which the zero order moment can be obtained)

"""

return (TS - TR)/(sig * np.sqrt(2*np.pi) * r) * (np.exp((-(np.log(r/rm))**2)/(2*sig**2)))

zero_moment = integrate.quad(zero_moment, 0, np.inf)[0]

def V(t):

a = 0.1

b = 18.1

rm = 33.9

r = (b * rm)/(rm + (b - rm)*np.exp(-a * t))

return (a * (1-(r/b)) * r)

#========================================================================================

**Appendix C: Python code for degradation term**

#%% import modules

import numpy as np

# Degradation term

def Z(t, c, d):

"""

Parameters

-----------------------------------------------------------------------------------------------------------------------------------------------------------

t : Time at point of measurement

c,d: Empirical coefficents from zero order moment

Returns

-----------------------------------------------------------------------------------------------------------------------------------------------------------

Degradation at time t

"""

return d * np.exp (c*t)

#========================================================================================

**Appendix D: Python code for drift term**

#%% Import modules

import numpy as np

# Drift term using moment analysis

def drift_term_moment(rm, sig):

"""

Parameters

-----------------------------------------------------------------------------------------------------------------------------------------------------------

sig: sigma

rm: median pore radius

Returns

-----------------------------------------------------------------------------------------------------------------------------------------------------------

Drift term (first order normalized moment)

"""

return rm * np.exp((sig**2)/2)

#========================================================================================

# Drift term using existing mathematical expression

def drift_term_expression(t, a, b, rm):

"""

Parameters

-----------------------------------------------------------------------------------------------------------------------------------------------------------

rm: median pore radius

a : temporal value of drift term

b : absolute value of drift term

t : Time at point of measurement

Returns

-----------------------------------------------------------------------------------------------------------------------------------------------------------

Drift term from existing mathematical expression

"""

r = (b * rm)/(rm + (b - rm)*np.exp(-a * t))

return (a * (1-(r/b)) * r)

#========================================================================================

# Cumulative drift term

def CT(f, a, b, N) :

"""

Parameters

-----------------------------------------------------------------------------------------------------------------------------------------------------------

f : Integrand (drift term function)

a,b: initial and final number of days of measurmeents

N : number of time steps

Returns

-----------------------------------------------------------------------------------------------------------------------------------------------------------

Cumulative drift term

"""

t = np.linspace(a, b, N)

ft = f(t)

CuT = np.sum(ft) * (b-a)/N

return (CuT)

#========================================================================================

**Appendix E: Python code for optimizing lambda (and obtaining the analytical solution)**

#%% import modules

import numpy as np

from scipy import integrate

# Fraction term with the exponential

M = integrate.quad(Z, 0, T)[0]

# Exponential term before the intergral

E = np.exp(M/T) # Exponential term

# Optimization of lambda

def f0(x):

"""

Parameters

-----------------------------------------------------------------------------------------------------------------------------------------------------------

TSi: initial measured saturated water content

TRi: initial measured residual water content

sigi: initial sigma

rmi: initial median pore radius

Returns

-----------------------------------------------------------------------------------------------------------------------------------------------------------

Initial pore size distribution

"""

return (TSi - TRi)/(x * sigi* np.sqrt(2*np.pi)) * (np.exp((-(np.log(x/rmi))**2)/(2*Si**2)))

def part1(x,a,b,lam):

"""

Parameters

-----------------------------------------------------------------------------------------------------------------------------------------------------------

a,b: integration variables

lam: lambda, the parameter to be optimized

Returns

-----------------------------------------------------------------------------------------------------------------------------------------------------------

Part of the analytical solution

"""

return ((1/(np.sqrt(4*b*lam*np.pi)) * ((np.exp(-((a-x+b)**2)/(4*b*lam))) + (np.exp((-a/lam) - (((a+x-b)**2)/(4*b*lam))))))) + ((1/(2*lam)) * np.exp(-a/lam) * special.erfc((a+x-b)/(np.sqrt(4*b*lam))))

def part2(x,a,b,lam):

"""

Parameters

-----------------------------------------------------------------------------------------------------------------------------------------------------------

a,b: integration variables

lam: lambda, the parameter to be optimized

Returns

-----------------------------------------------------------------------------------------------------------------------------------------------------------

Pieced together parts of the analytical solution

"""

return f0(x) * (part1(x,a,b,lam))

def I(a,b,lam):

"""

Parameters

-----------------------------------------------------------------------------------------------------------------------------------------------------------

a,b: integration variables

lam: lambda, the parameter to be optimized

Returns

-----------------------------------------------------------------------------------------------------------------------------------------------------------

Analytical solution (without the exponential part)

"""

II = integrate.quad(part2, 0, np.inf, args = (a,b,lam))[0]

return II

# Predicted values

def pred(r_meas,lam):

"""

Parameters

-----------------------------------------------------------------------------------------------------------------------------------------------------------

r_meas: Pore radius at which water retention parameters were measured

lam: lambda, the parameter to be optimized

Returns

-----------------------------------------------------------------------------------------------------------------------------------------------------------

Predictions for the optimization process

"""

PSD=[]

for i in range(len(r_meas)):

PS = I(r_meas[i], T, lam) # T is the cumulative drift term

PSD.append(PS)

return PSD

# Observed values to optimize lambda

# PSD is already defined in Fig. 1

# o stands for observed

fit = PSD(r_meas, TSo, TRo, sigo, rmo)

popt, pcov = curve_fit(pred, r_meas, fit) # Levenberg-Marquardt optimization

# popt gives the optimized value of lambda

#========================================================================================
